# Taking Stock of Security Concerns Related to Synthetic Biology in an Age of Responsible Innovation

**DOI:** 10.3389/fpubh.2014.00079

**Published:** 2014-07-09

**Authors:** Brett Edwards

**Affiliations:** ^1^Department of Politics, Languages and International Studies, University of Bath, Bath, UK

**Keywords:** synthetic biology, biosafety, biosecurity, ethics, dual-use, public health, policy

## Introduction

In early May, the German Ethics Council produced an in-depth report on the oversight of dual-use research of concern (DURC) (1). The report follows in the wake of recent international emergency reviews of avian influenza research and builds on discussions, which have been taking place internationally for over a decade (2).

In addition to calling for greater awareness raising and education in the scientific community, the report also calls for the establishment of a new legal framework to address DURC within Germany. This framework would provide a legal definition of DURC and would require researchers to report to a newly established central DURC committee before embarking on certain lines of research. Such a legal framework would also generate new responsibilities for those outside the research team who impact upon the research process; from funding right through to publication. For example, this would include new legal responsibilities for Laboratory Biosecurity Officers.

Such an approach would be in stark contrast to the patch-work of largely voluntary measures, which are in place in the rest of the world. The German Ethics Council has also taken the view that Germany should encourage the adoption of similar review models at EU level and internationally.

## Dual-Use and Precaution

The summary report of the full 300 page document produced by the German Ethics Council, which is yet to be published in English, notes that:

ethical analysis leads to the conclusion that scientific responsibility in the area of DURC is mainly to be governed by the *precautionary principle* (emphasis added) [Ref. (3), p. 3].

In essence, the precautionary principle places the burden of proof upon the scientific community to demonstrate that research of DURC should be carried out, and is being carried out in a responsible way. The precautionary principle tends to be brought into play in the context of complex risks, which are political challenges characterized by complexity, uncertainly, and ambiguity [Ref. (4), p. 235]. Dual-use issues are complex as they do not involve simple causal chains of events with easily quantifiable consequences, but rather a large set of intervening variables with unknown or even unknowable consequences. This is true not only in relation to thinking about the harms of research, but also its potential benefits. Dual-use issues also involve uncertainty, as there is insufficient data or information to convincingly produce risk verses benefit assessments of single experiments or lines of research. Dual-use issues are also ambiguous, as they typically involve conflicts over ethical and professional values.

Developing a legal and ethical framework to address these issues requires intricate webs of collaboration between institutions, in the context of policy strategy and design, as well as in the context implementation. This creates a challenging environment in which to develop and sustain policy initiatives, directed at problems, which receive only periodic interest from publics and governments.

The reassertion of the role of the precautionary principle in the context of dual-use research provides suitable moment to reflect on broader security concerns related to emerging techno-sciences such as synthetic biology, which extend beyond the single experiments commonly described as constituting DURC. These concerns relate to trends, which could undermine existing models of oversight (such as material and technology containment strategies). This includes concerns about the proliferation of foundation technologies, which could be utilized to modify or synthesize pathogens. These concerns also relate to broader trends in the underlying structures and funding of innovation (5). This includes concerns about de-skilling and proliferation dynamics in life-science research, which could potentially undermine existent and advocated approaches, which place emphasis on local level ethics review, as well as laboratory safety and security (6).

Many of these broader concerns are best thought of as *anxieties* rather than risks, in that discussions about them are largely speculative. However, non-proliferation experts have been keen to reassert, not only that new security challenges are inevitable, but also that existing national and international systems of oversight are poorly prepared (7). It is in this context that the field of synthetic biology has become somewhat of a test-bed for novel security initiatives. In the following section, there is an introduction to how dual-use concerns have emerged in relation to the broader field of synthetic biology, as well as some of the political realities facing those developing policy in this area.

## Emergence of Dual-Use Concerns about Synthetic Biology in a US and European Context

There have been discussions of security concerns related to the practices and technologies of synthetic biology, as far back as the community and institutions of the field can be identified. This is perhaps unsurprising, considering that the field emerged in the post 9/11 political environment. However, what is surprising is the high levels of attention this field has received as compared to other contemporary fields of innovation (such as nano-biotechnology).

A key reason for this is that engagement with misuse concerns has been a stipulation of research funding in both the US and the UK. The requirement to address dual-use concerns was incorporated into the National Science Foundation funding criteria for synthetic biology, when the first major publicly funded research center was established [Ref. (8), p. 15]. This led to the establishment of the first major ethical, legal, and social issue (ELSI) thrust with an explicit mandate to consider bioweapon issues. As a result, such concerns also took hold in a European context, as the field was being institutionalized by the research funding bodies. During these early stages a broad range of misuse concerns were under discussion, including those related to the threat of bioterrorism and biowarfare (9–11).

There are two key factors, which are important to thinking about dual-use as an ELSI issue. The first is that dual-use concerns have been a novel addition to more traditional ELSI concerns associated with new and emerging science and technology (such as safety). This means that the issue often competes with more established issues on the ELSI agenda. Security concerns have been more dominant in a US context, but less pronounced in a European ELSI context (12). Added to this, misuse concerns have emerged at a time in which the very concept and practice of ELSI governance is being made subject to transformation.

Both funders and society are increasingly demanding “up-stream” engagement by ELSI thrusts with the innovation process [Ref. (8), p. 15; Ref. (13)]. Up-stream engagement with the innovation process involves engineering safety and security into technologies and research practices, rather than just responding to the challenges raised by the products of innovation. Up-stream engagement is also typically understood to involve pro-active engagement with key regulators and stakeholders to pre-emptively address potential ethical and legal concerns. Increasingly, such engagement is understood as a part of national government policy to address forward looking concerns about new and emerging science and technology (14, 15).

However, there are several characteristics of dual-use politics, which de-limits the scope and feasibility of such endeavors, which have been reflected in the recent history of dual-use synthetic biology governance in a UK and US context.

## From Broad Anxieties to Narrow Actions

The first issue is that despite some of the regulatory back-lash myths, which linger in the US and Europe, governments have not tended to exhibit appetites to legislate *specifically* in relation to dual-use concerns related to synthetic biology. Particularly, with respect to those concerns, which could not be addressed through incremental amendments to law covering laboratory security and safety. Such a situation is symptomatic of a more general trend in dual-use governance in national contexts, in which there is an absence of clear institutional responsibility to develop such policy programs. It is worth noting, however, that even in the absence of “top-down” approaches, regulatory bodies can still play a fundamental role in the fate of so-called “bottom-up” initiatives; by providing financial, political as well as technical support. Such collaboration is also essential if up-stream engagement with the field is actually to result in the development, adoption, and sharing of best-practices nationally.

The second issue is that despite the emphasis on up-stream engagement and best practice sharing at institutions such as SynBERC there has not been substantial investment into systematic and nationwide examinations of the way in which dual-use issues are currently dealt with in different institutional contexts. This is even the case in relation to the field of synthetic biology. Such engagement is necessary if policy discussions about cutting-edge fields are going to be tied to concrete risk identification and management activities in the institutions in which research is taking place. It would seem that without such data gathering, much discussion, particularly in ELSI forum will be condemned to remain an exercise in “speculative ethics” (16).

A final issue is that while considering how to improve security practices at local level is important, there is still a requirement for institutional capacities to identify and respond to much more fundamental trends in S&T, which go beyond the scope of the local level review. In relation to synthetic biology in the US for example, the emphasis on the centrality of local level review, has led to an artificial narrowing of dual-use discussions. For example, the so-called “Sloan Report” (17) still represents one of the most substantial and influential technical reviews of security concerns related to synthetic biology. Yet this report largely externalized those concerns, which could not be identified and managed at local level. Other major reports produced in the US on synthetic biology have also tended to adopt this framing (14, 18).

In particular, the prospect of state level misuse of advances in the life-sciences in the development of weapons has been largely absent from US discussions of synthetic biology as a security concern. In a European context, the issue has only received substantial attention in more recent years. A report on an expert meeting hosted by the United Nations Interregional Crime and Justice Research Institute (supported by the European Commission), describes the various ways in which developments in synthetic biology could re-ignite military interest in biological weapons and potentially undermine existing oversight regimes at national and international level (19).

Such concerns are particularly pressing when one considers the existing challenges, which face the international regime tasked with preventing the development and use of biological weapons. This includes the absence of a system to verify state compliance, which is unlikely to change. While there is slightly more hope for improving the science and technology review system within the regime, improvements continue to be frustrated by a range of bureaucratic and diplomatic issues (20, 21).

## Conclusion

To sum up, the point of this article was not to argue that synthetic biology poses an imminent security threat, but instead to argue that while our capacity to imagine misuse scenarios is boundless, our institutional capacities to engage with the less whimsical of these concerns remains quite limited, and developments in policy in this area have been hard won. For some this will not be a cause for alarm, but for others, particularly those with less faith in the resilience of the current norm against biological weapons, this issue continues to be a source of unease.

## Conflict of Interest Statement

The author declares that the research was conducted in the absence of any commercial or financial relationships that could be construed as a potential conflict of interest.
